# Youths’ and Parents’ Experiences and Perceived Effects of Internet-Based Cognitive Behavioral Therapy for Anxiety Disorders in Primary Care: Mixed Methods Study

**DOI:** 10.2196/26842

**Published:** 2021-11-01

**Authors:** Josefine Lotten Lilja, Mirna Rupcic Ljustina, Linnea Nissling, Anna Caroline Larsson, Sandra Weineland

**Affiliations:** 1 Research, Development, Education and Innovation Primary Health Care Region Västra Götaland Göteborg Sweden; 2 Department of Psychology University of Gothenburg Gothenburg Sweden; 3 General Practice/Family Medicine, School of Public Health and Community Medicine Institute of Medicine, Sahlgrenska Academy University of Gothenburg Gothenburg Sweden

**Keywords:** internet, CBT, cognitive behavioral therapy, adolescents, parents, anxiety, primary care, mixed methods, experiences, youths, digital health

## Abstract

**Background:**

Anxiety is common among youths in primary care. Face-to-face treatment has been the first choice for clinicians, but during the COVID-19 pandemic, digital psychological interventions have substantially increased. Few studies have examined young people’s interest in internet treatment or the attitudes they and their parents have toward it.

**Objective:**

This study aims to investigate adolescents’ and parents’ attitudes toward and experiences of internet-based cognitive behavioral anxiety treatment in primary care and its presumptive effects.

**Methods:**

The study used mixed methods, analyzing qualitative data thematically and quantitative data with nonparametric analysis. Participants were 14 adolescents and 14 parents recruited in adolescent primary health care clinics. The adolescents and their parents filled out mental health questionnaires before and after treatment, and were interviewed during ongoing treatment.

**Results:**

The quantitative data indicated that the internet-delivered cognitive behavioral therapy program used in this study was successful in reducing symptoms (χ^2^_2_=8.333; *P*=.02) and that adolescents’ motivation is essential to the treatment outcome (*r*=0.58; *P*=.03). The qualitative results show that youths highly value their independence and freedom to organize treatment work on their own terms. The parents expressed uncertainty about their role and how to support their child in treatment. It was important for parents to respect the youths’ need for autonomy while also engaging with them in the treatment work.

**Conclusions:**

Internet treatment in primary care is accepted by both youths and their parents, who need clarification about the difference between their role and the therapist’s role. Patient motivation should be considered before treatment, and therapists need to continue to develop the virtual alliance. Finally, primary care should be clearer in informing adolescents and their parents about the possibility of internet treatment.

## Introduction

Cognitive behavioral therapy (CBT) is a well-documented and effective method for various states of anxiety and is considered the treatment of choice [1]. Furthermore, the Swedish National Board of Health and Welfare’s updated guidelines for depression and anxiety [2] recommend CBT before or at the same time as drug treatment for diagnosed conditions. However, access to CBT is limited for adults and children, and the COVID-19 pandemic has prompted a worldwide explosion in digital health interventions. The rapid adoption of digital psychological interventions such as internet CBT (iCBT) and video formats for therapy will certainly continue into the recovery from the pandemic and beyond. However, the recommendations for children and youth do not include iCBT [2] and few studies have examined young people’s interest in internet treatment or the attitudes they and their parents have toward it. This study aims to investigate adolescents’ and parents’ attitudes toward and experiences of internet-based anxiety treatment in primary care.

The effectiveness of internet treatment is comparable to that of in-person CBT [3] but with the advantages of greater accessibility, lower costs, and the potential for rapid dissemination and reaching patients who would otherwise not seek psychiatric care for fear of stigmatization [4,5]. Acceptance and commitment therapy (ACT) is considered a treatment method within the “third wave” of CBT. ACT aims to influence core processes maintaining various anxiety problems and is considered a transdiagnostic treatment. Internet-delivered ACT has been investigated in a systematic review that shows efficacy for anxiety disorders among adults [6], and a recent published study showed that acceptance-based iCBT was effective for adolescents with chronic pain [7].

There is a fast-growing research area examining iCBT for adolescents. There are studies on iCBT for children aged 8-12 years [8] and iCBT for those aged 13-19 years diagnosed with anxiety disorder [9,10]. However, in these studies the participants are recruited in response to website postings or local recommendations from health care centers, and none of them are conducted in the clinical context of routine primary care. Studies in clinical care are important to assess patients’ experiences and acceptance of treatment delivery.

Few studies have examined young people’s attitudes toward internet treatment. When Stallard et al [11] asked children and adolescents aged 8-17 years seeking help at a mental health clinic about their attitudes toward the internet or computer-based mental health programs, 25% of the answers were positive, 25% were negative, and 50% were indecisive.

Qualitative research on young people’s experiences of iCBT is also limited. Lenhard et al [12] interviewed 8 adolescents about their experiences of iCBT for obsessive-compulsive disorder (OCD) after treatment completion. Participants were recruited through advertisements in local newspapers, schools, and health care units in a metropolitan area. Results showed that young people appreciated being able to work independently; have control over the therapy process; have flexibility about time and space; be honest about their difficulties; and have the support of therapists, parents, and the content of the program [12]. Jones et al [13] found that caring adults constitute the most contributing factor when adolescents begin to seek help for their mental illness. After treatment begins, young people place more importance on the feeling of having control over their choices, which is associated with staying in treatment. The same study showed that youth’s perception of transparency in the therapeutic relationship is important for the treatment work itself. Getting suggestions as opposed to being told what to do contributed to their feeling of control, which in turn affected patients’ work with their symptoms [13]. Few studies have been conducted into young people’s experiences of provider contact in internet therapy. In their study of college students’ experiences of iCBT for generalized anxiety, Walsh and Richards [14] concluded that the development of “virtual alliance” is vital for client’s motivation to continue with iCBT.

Qualitative research into parents’ role, participation, and experience of internet treatment with their children is limited, and the field needs to be expanded. Spence et al [15] argue that if public health care aims to make internet treatment comparable to clinical treatment for children and adolescents, it needs to be accepted and approved by the parents, who usually initiate health care contacts for their children. The authors measured how satisfied young people and their parents were with internet treatment compared with clinical treatment. Both types of treatment were generally perceived as satisfactory by both groups. However, although there were no differences in the adolescents’ satisfaction, the parents were somewhat more satisfied with clinical treatment than with internet treatment [15].

According to Lundkvist-Houndoumadi et al [16], parental participation in CBT can vary based on two conditions. In one condition, parents are seen as cotherapists, who can facilitate generalized therapeutic learning through rewards, encouragement, praise, and other positive reinforcement. In the other, parents are more actively involved as copatients. They work simultaneously on their own feelings and behaviors as their children go into therapy, which can be an opportunity for both to work on the family dynamics that may contribute to adolescents’ anxiety problems [16].

In summary, little is known about how adolescents and their parents experience iCBT. The use of self-report instruments in previous studies may have limited their findings since their results might have too narrow a focus. For this reason, we aimed to gain a broader view of adolescents’ experiences of iCBT treatment for anxiety in primary care. To enable this broader understanding, youths’ and parents’ experiences were examined though their own stories in conjunction with self-reports on their well-being.

## Methods

This study was conducted in three Swedish primary health clinics, one in an urban area and two in suburbs. The implementation of iCBT was part of a research project conducted in 2017-2020 (Swedish National Research Register, FoU, ID 240221), approved by the Regional Ethics Committee in Gothenburg (Dnr 703-17).

### Study Design

This study used a mixed method convergent parallel design to examine an 8-week transdiagnostic iCBT program for adolescents with anxiety disorders treated in primary care. We used two methods to capture participants’ views of how the treatment had affected them, with the aim of grasping a deeper understanding of patients’ experiences than would be possible through only self-report or only interviews. The study thus used a convergent design, in which qualitative and quantitative data are intended to complement each other and elicit a richer understanding of the research problem [17]. In convergent designs the two types of data are collected during the same time frame and then compared. The quantitative and qualitative data in this study were thus collected during the same intervention period, with the intention to capture different dimensions of the experience. This is called the data diffraction approach [18]. Qualitative and quantitative data were analyzed separately, and we then integrated the analyses of the results to shed light on different aspects of the central phenomena through discussion. The qualitative data examined young people’s and their parents’ attitudes toward and experiences of iCBT. The study had a phenomenological approach (ie, initial analysis focusing on thorough descriptions, thereafter emphasis on interpretation being inherent in experience) and described the participants’ experiences of working with the treatment method [19,20]. The research approach was inductive, and the themes described were extracted from the data. In inductive analysis, data are encoded with no effort to fit them into an existing framework or according to the researcher’s analytical knowledge [19].

### Participants

Participants were 14 youths and one of their parents. The participants were recruited from three primary health clinics in the Västra Götaland Region on the west coast of Sweden. The inclusion criteria were mild to moderate anxiety problems such as social phobia, generalized anxiety disorder (GAD), panic syndrome, and unspecified anxiety syndrome. Exclusion criteria were severe or ongoing depressive episode, ongoing psychotherapeutic treatment or intervention study, alcohol or drug addiction, severe psychiatric symptoms requiring psychiatric care, risk of suicide, and neuropsychiatric disorder. Out of 14 participating youths, 9 (64%) were aged 13-15 years and 5 (36%) were aged 16-18 years (see Table 1 for demographic variables). The group was broadly representative of the economic and geographic diversity of the local population. Of the 14 child-parent pairs, all parents agreed to be interviewed. One female participant declined to be interviewed since she had not completed the assigned modules. Written informed consent to participate was obtained from all participating youths and parents.

**Table 1:** Demographic variables.

Variable			Participants (N=14), n (%)
**Age (years)**			
	13-15	9 (64)	
	16-18	5 (36)	
**Gender**			
	Boy	1 (7)	
	Girl	13 (93)	
	Other	0 (0)	
**Country of birth**			
	Sweden	14 (100)	
	Other	0 (0)	
**Parent’s country of birth**			
	Sweden	13 (93)	
	Other	1 (7)	
**Parent’s highest completed education**			
	Primary school	0 (0)	
	High school	3 (21)	
	University	11 (79)	
**Parent’s living situation**			
	Cohabitants	1 (7)	
	Married	11 (79)	
	Divorced/separated	1 (7)	
**Parent’s occupation**			
	Sick leave	1 (7)	
	Studying	1 (7)	
	Working	12 (86)	
**Years of current problem**			
	Less than a year	3 (21)	
	As long as I can remember	4 (29)	
	Other alternative	7 (50)	
**Previous psychological treatment**			
	No	3 (21)	
	Yes	11 (79)	
**Psychopharmacological medication**			
	Yes, current	0 (0)	
	Yes, terminated	0 (0)	
	No, never	14 (100)	

### Intervention

All participating youths received treatment through the iCBT program “Anxiety Help for Adolescents,” a guided internet-delivered self-help treatment program developed by Psykologpartners W&W AB. The intended treatment period is 8 to 12 weeks. “Anxiety Help for Adolescents” is a transdiagnostic program based on the principles of CBT for anxiety. Treatment interventions rely heavily on exposure therapy as described in a treatment manual developed by Hayes, Strosahl, and Wilson [21] and Hayes and Ciarrochi [22].

The iCBT program is aimed at young people between the ages of 13 and 19 with different anxiety diagnoses and is designed for the age and maturity of the targeted group. Theoretical concepts, clinical examples, and the overall structure of the digital treatment program have been exemplified and adapted for the target group through short videos, animations, and linguistic adaptation. The material is divided into eight different chapters/modules, with most participants expected to complete it in 10 weeks. Patients gradually learn new tools through exercises they can do independently, but the therapist is on hand to ask and answer questions and to follow up on the exercises through a messaging system within the program.

The therapists in the study were practicing in primary care in Västra Götaland in Sweden, working with psychological treatment of mental health problems in children and adolescents. The therapists were either licensed psychologists or psychologists under supervision before becoming licensed psychologists and had all been trained in the iCBT program “Anxiety Help for Adolescents.”

### Procedures

Young people (aged 13-18 years) seeking help at the primary health clinic for suspected anxiety issues and their accompanying parents were asked to participate in the study. A parent was present at assessment/inclusion and at follow-up talks. All patients were assessed in a clinical interview, and the structured interview MINI-KID (Mini International Neuropsychiatric Interview for Children) [23] was used at pre- and postassessment. The assessment interview was conducted by participating therapists, and the child-parent pairs completed all self-assessment scales for the premeasurements. The measurements used to assess treatment effects are listed in the following sections. All participants provided verbal and written consent prior to participation. Participants meeting the inclusion criteria were directed to iCBT treatment. After treatment, child-parent pairs met the treating psychologist for a final session to evaluate the outcome of therapy. In addition, re-evaluations were carried out according to MINI-KID, and the participants and parents completed all self-assessment scales for the postmeasurements.

Qualitative interviews were conducted continuously from spring and to autumn of 2019. Data were collected by clinical psychologists. The interviews lasted 30 minutes, were recorded using a digital voice recorder, and were transcribed verbatim. The qualitative interviews were conducted with patients and their parents when youths had completed a minimum of 6 modules. The patient group was to some extent homogeneous because they were recruited at the same type of clinics, sought help for anxiety problems, and underwent the same treatment. As the interviews were conducted and transcribed, response patterns began to repeat. The qualitative information was considered saturated and intake on the qualitative part stopped at 23 completed and transcribed interviews, 11 with young people, and 12 with one of their parents.

### Measurements for Youths

Self-assessment was performed upon inclusion (pretreatment), after the patients had completed two-thirds of the program (middle), and post treatment.

Symptoms of anxiety and depression in adolescents were measured with the Revised Children’s Anxiety and Depression Scale (RCADS) designed to assess clinical syndromes. The RCADS provides two total scores (anxiety and depression) and six subscales for separation anxiety disorder, social phobia, OCD, panic disorder, GAD, and major depressive disorder. The internal consistency of the RCADS subscales is high, with Cronbach α ranging from .78 to .88 [24,25].

General disability in young people was measured with the youth scale of the Education, Work and Social Adjustment Scale (EWSAS). The EWSAS measures adolescents’ general experienced level of functioning in school and social life [26,27]. It has an internally consistent construct across time with a near acceptable test-retest. The EWSAS also seems to relate to, though not directly measure, severity of illness and psychiatric disorder, and preliminary results support it as a sensitive measure of change for use among children and adolescents. The EWSAS is a valid and reliable assessment of functional impairment that is easy and quick to administer in both research and clinical settings [27].

Global functioning was measured with Children’s Global Assessment Scale. The interviewer assesses the patients’ level of functioning on a scale of 1 to 100, with a higher score indicating a better or higher level of life functioning [28].

Acceptance/psychological flexibility in young people was measured with the Avoidance and Fusion Questionnaire Youth (AFQ-Y8). AFQ-Y8 may be a valuable clinical tool in reflecting changes in psychological flexibility among adolescents aged 12-18 years [29].

Motivation for treatment was measured with the Nijmegen Motivation List 2 (NML-2) [30]. The instrument was designed to measure patient motivation for CBT. The NML-2 consists of three factors: preparedness, distress, and doubt. Preparedness expresses the patient’s preparedness to actively invest in treatment and to make sacrifices. Distress expresses how the patients’ health negatively affects others and themselves. Doubt expresses the patient’s uncertainty about their investment in treatment, the treatment itself, and the possibility of gaining from it. The NML-2 total scores were associated with proximal-treatment helpfulness and with treatment dropout. Higher scores on the NML-2 (range 0-30) reflect higher motivation for treatment. Internal consistency and retest reliability of the factors have been shown to be reasonable [30].

### Measurements for Parents

Symptoms of anxiety and depression in adolescents were measured with the Revised Child Anxiety and Depression Scale-Parent (RCADS-P), which assesses parents’ reports of youths’ symptoms of anxiety and depression across the same six subscales as the RCADS previously described. The RCADS-P can be used to track symptoms and provide additional information for assessment [24,25].

General disability in young people was measured with the EWSAS-parent scale. The EWSAS-parent assesses parental reports of youths’ levels of general disability.

Perceived parental stress was measured with the Hospital Anxiety and Depression Scale (HADS). The HADS [31,32] consists of 14 statements (7 on depression and 7 on anxiety) with four response alternatives (0-3). The HADS has been shown to be a reliable and valid instrument for the detection of anxiety and depression in individuals from 16 to 65 years of age [26]. Its reliability was shown by Herrmann [33] with Cronbach α on HASD-A at .80 and on HADS-D at .81. The maximum score on each subscale is 21, and 11 points is the cutoff level for a diagnosis of anxiety or depression. Values of 0 to 6 indicate no or normal anxiety or depression [31].

Motivation for treatment was measured with the NML-2 parent. The NML parent assesses parental reports of youths’ motivation to engage in CBT.

### Interviews

Semistructured interview guides consisted of questions about experiences and expectations of the treatment before it began and during the treatment, of the treatment interventions themselves, the contact with the therapist, and thoughts about the future after the treatment was completed. Two interview templates were used, one for adolescents and one for parents. The questions were open-ended to facilitate reflection, and probing questions were asked to elicit further exploration [34].

### Data Analysis

#### Quantitative Data Analysis

Quantitative data analysis was performed using SPSS Statistics 25 (IBM Corp). The quantitative premeasurement, middle, and postmeasurement data for the youths were analyzed using a nonparametric statistical method for repeated measures, Friedman analysis of variance (ANOVA) [35,36]. The nonparametric Friedman ANOVA was used because of the small sample size and the assumption of nonnormality of data. The nonparametric test Wilcoxon signed rank test for related samples was used for posthoc analysis. In the last step, Pearson correlation coefficient was used to assess the relationship between motivation for treatment (scored by NML-2; N=14) and changes in symptoms of anxiety and depression (assessed by RCADS-Total and RCADS-Anxiety) between pre- and postintervention. Wilcoxon signed rank test for related samples were used to analyze the parents’ pre- and postintervention scores.

#### Qualitative Data Analysis

Data, in this case transcripts of interviews, were analyzed using thematic analysis. Thematic analysis, as defined by Braun and Clarke [19], is a method for identifying, analyzing, and reporting patterns or themes in data as an aid to their organization and description. Thematic analysis is argued to be a flexible and useful research tool because of its theoretical freedom [19].

The data were thematically analyzed using the six steps proposed by Braun and Clarke [19]. In the first step, the material was read carefully and repeatedly to help researchers become familiar with the content as a whole. In the second step, data were coded according to their interesting aspects in relation to the research questions. Examples of codes include “time” or “difficulties with the internet.” In the third step, all code names were collected under common subthemes that described repeated patterns in the responses, such as “treatment work” or “the therapist via the network.” In the fourth step, a few themes were developed and analyzed against the entire database. In the fifth step, a concrete thematic map was created with four main themes and several subthemes. In the sixth and final stage, themes were linked both to research questions about attitudes toward and experiences of iCBT treatment and to relevant research on these issues. The first and second authors (JLL and MRL) interpreted the data dialectically, moving between their preunderstandings and the data, and these interpretations were discussed until a consensus was reached on the formulation of the themes presented here. The analysis was repeated by the second and last authors (MRL and SW) to ensure reliability/trustworthiness. Disagreements were resolved through discussion and consensus. The NVivio12 (QSR International) computer program was used as support in data processing.

During the analysis, we considered and reflected upon the ethical aspects raised by Malterud [37]: reflexivity around our preunderstandings and meta-positions, transferability of the data from the selected sample, for whom or what the results are relevant, and the interpretation and analysis of the date, including theoretical preferences and transparency of the procedures. The authors’ considered their preunderstandings, and upon ethical reflection, only the third author (LN) had a small clinical experience of working with patients in the treatment program Anxiety Help for Adolescents. We believe our approach to the data, interpretation, and analysis were neutral, as we had no expectations or preunderstandings of the participants’ answers to questions about the method or their participation in internet treatment. At the same time, for the past 3 to 8 years, all authors have used CBT with adolescents and adults in primary and psychiatric health care. This professional experience has created an in-depth knowledge and positive attitude toward CBT and its clinical application, which could have influenced the analysis.

## Results

### Quantitative Results

The quantitative results are based on the 7 adolescents and 9 parents that completed the pre-, middle, and postmeasurement. Tables 2 and 3 show the results for the participating adolescents and parents.

**Table 2:** Results for participating youths on outcome variables RCADS, AFQ-Y8, EWSAS, and NML-2.

Variable			Mean (SD)		Participants
**RCADS^a^ total score**					
	Pre	70.21 (7.6)		14	
	Middle	66.00 (7.3)		11	
	Post	59.00 (11.8)		8	
**RCADS-Anxiety**					
	Pre	69.93 (7.9)		14	
	Middle	64.73 (6.3)		11	
	Post	57.88 (11.0)		8	
**AFQ-Y8^b^**					
	Pre	18.79 (5.8)		14	
	Middle	17.09 (5.0)		11	
	Post	16.25 (8.4)		8	
**EWSAS^c^**					
	Pre	16.79 (6.0)		14	
	Post	18.25 (6.5)		8	
**NML-2^d^**					
	Pre	87.29 (6.5)		14	

^a^RCADS: Revised Children’s Anxiety and Depression Scale.

^b^AFQ-Y8: Avoidance and Fusion Questionnaire Youth.

^c^EWSAS: Education, Work and Social Adjustment Scale.

^d^NML-2: Nijmegen Motivation List 2.

**Table 3:** Result for participating parents on outcome variables RCADS, EWSAS, and HADS.

Variable		Mean (SD)	Participants, n
**RCADS^a^ total score**			
	Pre	73.14 (7.4)	14
	Post	61.44 (9.7)	9
**RCADS-Anxiety**			
	Pre	71.43 (8.6)	14
	Post	60.44 (9.8)	9
**EWSAS^b^**			
	Pre	10.93 (6.0)	14
	Post	5.22 (5.0)	9
**HADS^c^ total score**			
	Pre	10.57 (5.2)	14
	Post	10.67 (5.9)	9
**HADS-Anxiety**			
	Pre	7.57 (3.0)	14
	Post	6.67 (3.6)	9
**HADS-Depression**			
	Pre	3.00 (2.8)	14
	Post	4.00 (2.6)	9

^a^RCADS: Revised Children’s Anxiety and Depression Scale.

^b^EWSAS: Education, Work and Social Adjustment Scale.

^c^HADS: Hospital Anxiety and Depression Scale.

### Participating Youths With Complete Data (n=7)

#### RCADS Total

The results from the Friedman test for the RCADS Total score showed that there was a statistically significant difference between measurement points (χ^2^_2_=8333; *P*=.02). Post hoc analysis with the Wilcoxon signed rank test for related samples showed a statistically significant reduction in the 7 youths’ total scores on anxiety and depression symptoms from pre- to postintervention (*Z*=−2.201; *P*=.03; *r*=0.83).

#### RCADS-Anxiety

The results from Friedman test for RCADS Total Anxiety score showed a statistically significant difference between measurement points (χ^2^_2_=9.652; *P*=.008). Post hoc analysis with the Wilcoxon signed rank test for related samples showed a statistically significant reduction in the 7 youths’ total anxiety symptoms from pre- to postintervention (*Z*=−2.207; *P*=.03; *r*=0.83).

#### EWSAS

The results from the Wilcoxon signed rank test for related samples on the EWSAS showed no statistically significant difference between pre- and postintervention (*Z*=−0.677; *P*=.50; *r*=0.26).

#### AFQ-8

The results from the Friedman test for the AFQ-8 showed no statistically significant difference between measurement points (χ^2^_2_=0.560; *P*=.76). No post hoc analysis was performed.

### Perceived Parental Stress: Parents With Complete Data (n=9) and Their Scoring of Their Children’s Symptoms

#### RCADS Total

The results from the Wilcoxon signed rank test for related samples for the RCADS Total score for the parents showed a statistically significant difference on the parents scoring of their children’s symptoms on anxiety and depressive symptoms between pre- and postmeasurement (*Z*=−2.521; *P*=.01; *r*=0.84).

#### RCADS-Anxiety

The results from the Wilcoxon signed rank test for related samples for the RCADS Total Anxiety score for the parents showed that there was a statistically significant reduction in how the parents scored the children’s total anxiety symptoms between pre- and postintervention (*Z*=−2.668; *P*=.008; *r*=0.89).

#### EWSAS

The results from the Wilcoxon signed rank test for related samples on the parents scoring on the EWSAS showed a statistically significant improvement of the children’s general functioning between pre- and postintervention (*Z*=−2.077; *P*=.04; *r*=0.69).

### Perceived Parental Stress: Parents With Complete Data (n=9)

#### HADS

The results from the Wilcoxon signed rank test for related samples on the parents scoring of their symptoms on the HADS (total score) showed no statistically significant difference between pre- and postintervention (*Z*=−0.535; *P*=.59; *r*=0.18).

#### HADS-A

The results from the Wilcoxon signed rank test for related samples on the parent’s symptoms on anxiety showed no statistically significant difference between pre- and postintervention (*Z*=−1.786; *P*=.07; *r*=0.60).

#### HADS-D

The results from the Wilcoxon signed rank test for related samples on the parents scoring of their symptoms of depression showed no statistically significant difference between pre- and postintervention (*Z*=−0.948; *P*=.34; *r*=0.32).

### Relationship Between Motivation and Changes in Symptoms of Anxiety and Depression

The results of the analysis with the Pearson correlation coefficient showed a statistically significant relationship between motivation for treatment, assessed by NML-2 and scored by the participating adolescents before the start of the treatment, and changes in the RCADS-Total score for anxiety and depression (*r*=0.58; *P*=.03) between pre- and posttreatment. Moreover, there was a statistically significant strong relationship between motivation for treatment, assessed by NML-2 scored by the participating adolescents, and changes in scores for RCADS subscale Anxiety between pre- and postintervention (*r*=0.63; *P*=.02).

The analyses using the Pearson correlation coefficient between the participating parents’ scoring of their children’s motivation for treatment, as assessed by the NML-2 before the start of treatment, and their scoring of their adolescents’ changes on RCADS between pre- and posttreatment showed no statistically significant results for either the RCADS-Total (*r*=0.52; *P*=.06) or the subscale for anxiety (*r*=0.49; *P*=.07). Moreover, there was no statistically significant relationship between the parents’ scores of their adolescents’ motivation for treatment and changes of RCADS when scored by the adolescents themselves between pre- and posttreatment for either the total scale (*r*=0.37; *P*=.19) or the subscale for anxiety (*r*=0.43; *P*=.12).

### Qualitative Analysis

Thematic analysis of the 11 interviews with adolescents and 12 interviews with parents resulted in four overarching themes and several subthemes. The results are presented in Textbox 1 and illustrated in the text with quotations.

**Presentation of overarching themes and subthemes.**

1. Breaking new grounds

1.1. Adolescents: positive yet uncertain attitudes

1.2. Parents: an ambivalent attitude

2. The adolescent behind the wheel

2.1. Adolescents: needs to be individualized

2.2. Adolescents: an independent task

2.3. Adolescents: a varied relationship with the therapist

2.4. Parents: program requires the youths’ independence

3. The role and function of parents

3.1. Adolescents: parents have a reminding and supportive function

3.2. Parents: limited insight into treatment

4. The effects of treatment

4.1. Adolescents: increased knowledge

4.2. Parents: increased understanding and changed behaviors in the youths

4.3. Parents: concerns about the future

### Breaking New Ground

#### Adolescents: Positive Yet Uncertain Attitudes

All adolescents in the study, regardless of their previous experience with psychological treatment, described being offered treatment on the internet as something new. Most young people described feeling uncertain about what it would mean to work with their mental health via the internet. Several said they were offered internet treatment at their health unit as an alternative and that they saw it as an opportunity to get help faster, which contributed to a more neutral and positive attitude:

I was a little hesitant. It felt strange to think that a programme on the net could help me like [...]. Then it felt good because I, ah, I had come so far that I had, like, sought help.

Youth 3

#### Parents: An Ambivalent Attitude

Most parents in the study had not known about the possibility of treatment via the internet, but all of them said they saw it as an opportunity to at least start to get help for their child. However, most expressed skepticism about whether the treatment would work since there would be no in-person, face-to-face contact with the therapist. Some parents wondered whether and how the treatment would work if the child had to complete it alone, but all were positive about trying it:

It could also be something that maybe you should think about. Do I fix this via the Internet or set the goals on my own? For some it may work better if you have a personal contact, and you get a task to solve for the next time. I think that is a little bit...you should probably check how I work in such a context. Am I fixing to do this myself or is it good to have this personal contact?

Parent 1

### The Adolescent Behind the Wheel

#### Adolescents: Need to Be Individualized

The young people in the study were consistently positive about the treatment program and would recommend it to others. They described various components, tools, and metaphors from the program that they had been thinking about or had worked with. Adolescents appreciated how the program alternated between text, pictures, and films, and that several people with different anxiety problems were presented in the program. However, several young people said they wanted the program to be even more individualized. Some experienced the program as time limited, while others believed that more log-ins would have helped to keep their work with the program more consistent:

It’s great that it’s not all text on one page, but that you browse and that it’s new text. I have thought about that. That’s really good. Because otherwise, it would be much more boring, I think. It’s a lot of pictures and so on, and a lot of videos. It’s good.

Youth 6

#### Adolescents: An Independent Task

An important aspect that all young people in the study raised was the independence of the treatment work. They appreciated this partly because they did not need to involve their parents and partly because it was just their own. Most highlighted their ability to keep the treatment work to themselves as an important positive experience. Another advantage of this independence was the opportunity to work when and how it suited them. The young people described how they worked on the treatment on their own and that it was up to them to formulate goals and implement changes at their own pace. Several described how they adapted their time or work with the treatment to fit in with other demands in their life. Being able to pause the treatment or adjust when they worked with it to continue to meet their school’s requirements was an important benefit for most young people. However, most young people also described the disadvantages of working via the internet. In many cases, they lacked confidence in their own ability to work therapeutically via the internet:

There are still those kinds of things that I – like the programme is not doing it. It helps help me, so that I can see everything, how I should do it, but I am still the one who has to do everything.

Youth 9

#### Adolescents: A Varied Relationship With the Therapist

In general, the adolescents in the study said they were satisfied with their contact with the therapist, even those who did not have much contact. Some youth had contact on a regular basis, while some had no interest in having contact even if they were aware of the opportunity. Those who were in contact with the therapist described receiving help to individualize their goals or support regarding the program itself. Some young people said they did not know what kind of support they could get from the therapist:

It is not as good contact as when you had...as if you had talked to them in person, but it is still a very good contact.

Youth 1

#### Parents: Program Requires the Youths’ Independence

Most parents in the study had limited insight into their child’s treatment work, although most knew that the child was doing that work. Most also knew that there was contact with the therapist during the treatment. At the same time, few parents knew much about how their child arranged the treatment work or what the contact with the therapist looked like. Parents generally expressed respect for the children’s treatment work, and many described their children as competent, dutiful, and capable individuals. Parents consistently appreciated their children for their commitment and participation in the treatment:

Since she did not want me to sit beside her when she did it, I had to accept it because she is so big that, yes, yes, she has to choose for herself whether I should participate or not, I feel.

Parent 5

### The Role and Function of Parents

#### Adolescents: Parents Have a Reminding and Supportive Function

The adolescents in the study described how their parents were a welcome support when they initially sought help, contacted health care, and awaited treatment but became less involved during treatment. The youth described feeling supported by their parents, who they perceived would be available if they needed help:

So, it was maybe that my mom kind of tried to talk about it with me. But it was more like I felt it was not a good idea to talk about it.

Youth 7

#### Parents: Limited Insight Into Treatment

The parents in the study reflected on their parental role, not only in their child’s treatment but also in general. All parents had a clear appreciation of their children, their characteristics, their anxiety problems and how they developed, and their bravery in seeking help. The parents saw the treatment as aimed toward the child but were unclear about expectations around their own participation. All parents in the study said that they left control over the level of their own participation in the treatment work to their child. Several saw themselves as supporters even though they felt outside the treatment itself, which contributed to their uncertainty about their own role. Some parents wondered whether learning more about the content of the treatment would help them to support their child. Most parents had reflected on the dilemma of how to relate to and support youths expressing their independence while also meeting their needs for support and assistance in treatment. All parents in the study discussed having reflected on the balances between proximity and distance, nagging or stepping back, and staying close but not too involved:

I mean I would also like to keep track of things, but I had to...I mean it’s like no toddler I have to deal with. She’s about to grow up and somehow has to know, and [I have] to show that “I believe in you fixing this”. So, I’m worried I can’t directly say. I’d say I’m rather a bit more curious about what she has done.

Parent 3

### The Effects of Treatment

#### Adolescents: Increased Knowledge

The young people reflected on what they had learned in the treatment about their problems and how they could handle them in the future. Everyone described a process of change from the time they had been offered the treatment until the interviewer called them. On whether the therapy led to improvement or whether their anxiety was still perceived as problematic, all said that they had learned more about their anxiety and how they could handle it differently in the future:

Like, if it’s something I really don’t want to do, then maybe I’m thinking about something I’ve learned there, that it’s better to do it, otherwise you get long-term problems and then, it gets easier. Then you do it.

Youth 5

#### Parents: Increased Understanding and Changed Behaviors in the Youths

All parents in the study noticed changes in how their children handled anxiety. Most described how their children’s own understanding of their problems increased over the course of the treatment, and some also saw changes in their behaviors:

So, she’s gone out to do things I couldn’t dream of her doing.

Parent 4

#### Parents: Concerns About the Future

The parents expressed uncertainty about whether the changes they noticed could be attributed to the treatment or to the children’s natural development and maturation. Some also expressed concern about what might happen in the future if the child got worse and highlighted the importance of their being able to return to the program to keep the knowledge alive:

No, but I really think that as long as the programme continues, then it’s going to...then you are reminded if you forget it, and so there is probably no worry. But what I think of, what I started with, is what is there left once you’ve finished it?

Parent 6

## Discussion

### Principal Findings

The purpose of the study was to investigate in adolescents and their parents their attitudes to and experiences of working with iCBT for anxiety problems. We chose a mixed-methods design to enable a deeper understanding of patients’ experiences than would be possible through only one method. The study focused mainly on participants’ experiences during the treatment but also highlighted their expectations of iCBT and its presumptive effects.

The quantitative data showed that the youths’ symptoms of anxiety and depression improved after completing treatment. These results indicate that the iCBT program was successful in reducing symptoms, which aligns with prior research showing that iCBT is an effective treatment method for adolescents [8,38]. These quantitative results also align with the qualitative results of this study, in which the participating adolescents described how the treatment increased their knowledge and contributed to altering views about their own anxiety problems.

The parents also assessed their children’s general functioning as better post treatment, which aligns with the qualitative results in which the parents perceived how their children managed their anxiety problems in a different way. At the same time, parents also expressed concerns that the changes might be short-lived.

The quantitative results further showed a strong relationship between the participants’ initial motivation to treatment and outcome. It is possible that youths with higher motivation for treatment before starting treatment also engaged more fully in the iCBT program, which most likely would have affected their treatment outcomes. Several studies on attitudes to internet-delivered psychological treatments highlight the benefits of such treatment (eg, increased ability to work independently and control over the therapy process) [12,39,40], but findings suggest that iCBT treatment might also place more responsibility, and hence a burden that could exacerbate anxiety, on the patients. It is possible that higher initial motivation for treatment increases the ability to structure one’s own time and create favorable conditions for engaging in the treatment program. Initial motivation for iCBT treatment might thus be an important factor for the clinician to explore before initiating iCBT treatment with patients in primary care.

The results showed no statistically significant relationship between how parents assessed their adolescents’ motivation for treatment and any changes in their symptoms of depression and anxiety as rated by both patients and parents on the RCADS-Total and RCADS-Anxiety. Because of the limited sample size, no major conclusions should be drawn from this, but it is an interesting finding from a clinical perspective. For a clinician, it may be more important to explore and consider the child’s motivation, rather than the parent’s perception, before deciding to initiate an iCBT treatment. Parents often have opinions about appropriate and preferable treatments for their children, but the results of this study indicate that parents’ perceptions of their child’s motivation for a particular treatment might have little to do with the child’s outcome in therapy. The child’s own motivation for treatment seems to be more important than their parents’ assumptions and to have a greater association with the treatment outcome.

Both adolescents and their parents described a generally positive attitude toward help with mental health problems via the internet and saw iCBT as an acceptable treatment alternative. The study’s results are comparable to those of previous studies that have shown a variation from neutral to positive attitudes to iCBT among youths [15]. The youths in our study expressed a positive attitude to the treatment and would recommend it to other young people with anxiety problems. They described having learned about their own anxiety no matter how successful they felt the treatment had been for them. Contact with the therapist during treatment was perceived as small but sufficient in this study, and the therapist was described as friendly and supportive. Similar to other features of the internet treatment, even contact with the therapist was perceived as having been conducted on the young people’s terms.

Parents described positive changes in their adolescents’ knowledge and management of anxiety, but they also had concerns that these effects might be short-lived and disappear after treatment completion. The parents’ insights into their children’s treatment work and contact with the therapist were limited. Parents saw their children as working independently and in not much need of parental support when working with iCBT. The parents tried to respect and acknowledge their growing children’s need for independence and autonomy, but also wanted to be supportive of the treatment work and were uncertain about how to help without being intrusive.

Previous research on parental involvement in adolescents’ internet therapy has shown the importance of the role of parents in introducing internet therapy to patients younger than 18 years while recognizing that their importance decreases as treatment continues [13,15,16]. However, previous research did not include parents’ perceptions of their own participation and role during their children’s participation in iCBT. This study’s results show that parents vary in how much and in what way they wish, or are able, to be involved in their children’s internet treatment. On the one hand, they want to know more about the content of the treatment program to be able to better support their children; on the other hand, they want to let the young people themselves control their treatment work. Despite this contradiction, parents described how they reminded, nagged, and asked about the treatment program, consistent with the role of the therapist in adult iCBT who reminds, motivates, and helps with structures [8].

The study’s findings on youths’ and their parents’ experiences of treatment and the youths’ experiences of contact with the therapist could contribute to answering the question raised by Vigerland et al [8] about how a division of roles between parents and caregivers could function in youth therapy via the internet. Through their role as someone who supports, reminds, and is on hand in everyday life, parents could take on the role of cotherapists and thus take over part of the therapist role. The virtual alliance with the therapist could then focus more on increasing compliance and individualizing the internet treatment, and less on motivating the youth to remain in treatment. As noted by Badawy and Radovic [41], a number of challenges and further research is needed to improve telemedicine and iCBT that is offered to young people. Optimizing digital approaches to health care delivery and integrating them into the public health will continue during the current COVID-19 outbreak and other future worldwide crises. In this, it will be important to analyze quality of care with feedback from patients and health care providers as well as cost-effectiveness, degree of improvement of mental health, and balance in use.

In summary, this study’s results support the importance of parents’ involvement as an important part of iCBT work with young people. This applies not only at the start of treatment as found by Jones et al [13], but also throughout the treatment. Informing and introducing parents to iCBT and the expectations of their participation, and supporting their collaboration with therapists can create even better conditions for the young people undertaking iCBT treatment.

### Limitations

The sample in the study was restricted to a gender-biased and small sample of young people and adults, which limit the generalizability of the results but challenges future research to investigate other experiences of internet treatment. The gender bias is important to address in future research and clinical work. We need more generalized data and improved ways to reach boys in early stages of mental illness in primary care. The interviews in the study were conducted during treatment, which could affect the results, as participants may feel compelled to express more positive attitudes than would be the case if the interviews were conducted after completion of treatment or at a later date.

### Conclusions

This study’s unique contribution about the practical benefits of iCBT for youths is its implementation in a primary care context. The results provide further support for offering internet treatment as a firsthand option to youth seeking mental health care at primary care units. Internet treatment should primarily be offered to motivated young people who have expressed a need to control their own time and those who want to work with psychological treatment independently and without eye-to-eye contact with their therapist.

The take-home messages for clinicians and health care organizations in primary care can be summarized as follows:

- Youths prefer a therapist who they perceive as one who can both give “support” and provide shared reflective opportunities. This finding speaks to maintaining a fundamental emphasis on a relational approach; in other words, for a therapeutic relationship that places the experience of human contact and response in the forefront, whether that experience be digital or physical.
- Youths and parents treated in primary care generally have a positive attitude and experience of iCBT during treatment.
- The participant’s motivation should be considered before initiating treatment.
- The parent’s role and involvement in iCBT throughout therapy needs clarification when initiating treatment.

## Abbreviations

ACT: acceptance and commitment therapy
AFQ-Y8: Avoidance and Fusion Questionnaire Youth
ANOVA: analysis of variance
CBT: cognitive behavioral therapy
EWSAS: Education, Work and Social Adjustment Scale
GAD: generalized anxiety disorder
HADS: Hospital Anxiety and Depression Scale
iCBT: internet cognitive behavioral therapy
MINI-KID: Mini International Neuropsychiatric Interview for Children
NML-2: Nijmegen Motivation List 2
OCD: obsessive-compulsive disorder
RCADS: Revised Children’s Anxiety and Depression Scale
RCADS-P: Revised Child Anxiety and Depression Scale-Parent
