# Correction: Youths’ and Parents’ Experiences and Perceived Effects of Internet-Based Cognitive Behavioral Therapy for Anxiety Disorders in Primary Care: Mixed Methods Study

**DOI:** 10.2196/35350

**Published:** 2021-12-02

**Authors:** Josefine Lotten Lilja, Mirna Rupcic Ljustina, Linnea Nissling, Anna Caroline Larsson, Sandra Weineland

**Affiliations:** 1 Research, Development, Education and Innovation Primary Health Care Region Västra Götaland Göteborg Sweden; 2 Department of Psychology University of Gothenburg Gothenburg Sweden; 3 General Practice/Family Medicine, School of Public Health and Community Medicine Institute of Medicine, Sahlgrenska Academy University of Gothenburg Gothenburg Sweden

In “Youths’ and Parents’ Experiences and Perceived Effects of Internet-Based Cognitive Behavioral Therapy for Anxiety Disorders in Primary Care: Mixed Methods Study” (JMIR Pediatr Parent 2021;4(4):e26842) the authors noted one error.

In the originally published manuscript, some affiliations were missing for first author Josefine Lotten Lilja. Only affiliation 1 was listed for this author, but all 3 affiliations on the paper should have been listed for this author.

The full list of authors and affiliations was originally published as follows:

Josefine Lotten Lilja^1*^, PhD; Mirna Rupcic Ljustina^1^, PsyM; Linnea Nissling^1,2,3*^, PsyM; Anna Caroline Larsson^1*^, PsyM; Sandra Weineland^1,2,3*^, PhD

^1^Research, Development, Education and Innovation, Primary Health Care, Region Västra Götaland, Göteborg, Sweden

^2^Department of Psychology, University of Gothenburg, Gothenburg, Sweden

^3^General Practice/Family Medicine, School of Public Health and Community Medicine, Institute of Medicine, Sahlgrenska Academy, University of Gothenburg, Gothenburg, Sweden

^*^these authors contributed equally

The list of authors and affiliations has been corrected as follows:

Josefine Lotten Lilja^1,2,3*^, PhD; Mirna Rupcic Ljustina^1^, PsyM; Linnea Nissling^1,2,3*^, PsyM; Anna Caroline Larsson^1*^, PsyM; Sandra Weineland^1,2,3*^, PhD

^1^Research, Development, Education and Innovation, Primary Health Care, Region Västra Götaland, Göteborg, Sweden

^2^Department of Psychology, University of Gothenburg, Gothenburg, Sweden

^3^General Practice/Family Medicine, School of Public Health and Community Medicine, Institute of Medicine, Sahlgrenska Academy, University of Gothenburg, Gothenburg, Sweden

^*^these authors contributed equally

The correction will appear in the online version of the paper on the JMIR Publications website on December 2, 2021, together with the publication of this correction notice. Because this was made after submission to PubMed, PubMed Central, and other full-text repositories, the corrected article has also been resubmitted to those repositories.

